# Virale Hepatitis – ein versteckter Killer

**DOI:** 10.1007/s00103-021-03485-9

**Published:** 2022-01-11

**Authors:** Eberhard Hildt

**Affiliations:** grid.425396.f 0000 0001 1019 0926 Abt. Virologie, Paul-Ehrlich-Institut – Bundesinstitut für Impfstoffe und biomedizinische Arzneimittel, Paul-Ehrlich-Str. 51–59, 63225 Langen, Deutschland

Die virale Hepatitis, eine entzündliche Erkrankung der Leber, stellt weltweit gesehen eines der großen Probleme für das Gesundheitswesen dar. In der Zeit der SARS-CoV-2-Pandemie steht naturgemäß eine andere virale Erkrankung im Vordergrund, aber auch in anderen Zeiten scheint es aus europäischer Perspektive bisweilen in Vergessenheit zu geraten, dass weltweit mehrere Hundert Millionen Menschen von einer viralen Hepatitis betroffen sind und die Erkrankung häufig mit starker gesundheitlicher Belastung und hoher Morbidität einhergeht.

Die meisten Fälle einer viralen Hepatitis sind durch 5 biologisch völlig unterschiedliche Viren bedingt: die Hepatitisviren A, B, C, D und E (HAV–HEV). Insbesondere chronische HBV- und HCV-Infektionen sind mit schwerer Morbidität und Mortalität assoziiert. Weltweit leiden derzeit ca. 257 Mio. Menschen an einer chronischen HBV-Infektion und ca. 71 Mio. Menschen an einer chronischen HCV-Infektion. Davon versterben ca. 1,4 Mio. Menschen jährlich an den Folgen einer Infektion mit HBV oder HCV. Grund genug, sich intensiv mit der Epidemiologie, der molekularen Virologie, Pathogenese, Therapie und Prävention der viralen Hepatitis auseinanderzusetzen.

Das vorliegende Themenheft „Virale Hepatitiden in Deutschland“ befasst sich mit dieser Thematik mit einem Fokus auf die Situation in Deutschland.

Im ersten Beitrag, verfasst von *Bender et al.*, werden die im Themenheft behandelten Viren zunächst vorgestellt. Dabei werden ihre Phylogenie, ihre Genomstruktur, ihr Lebenszyklus und spezielle Aspekte ihrer Pathogenese beschrieben. Obgleich es sich um phylogenetisch grundverschiedene Viren handelt, weisen sie insbesondere hinsichtlich der virusassoziierten Pathogenese Gemeinsamkeiten auf, die – wie in dem Beitrag beschrieben – teilweise jedoch durch sehr unterschiedliche Mechanismen zustande kommen.

Entsprechend dem Titel des Themenheftes „Hepatitiden in Deutschland“ beleuchtet der Beitrag von *Dudereva et al.* die Epidemiologie der Virushepatitiden A bis E in Deutschland. Der Beitrag beschreibt dabei auch die in Deutschland relevanten Übertragungswege für diese Erreger und lenkt das Augenmerk bei HEV auf die zoonotische Übertragung. Weiterhin werden die in Deutschland verfolgten Therapieansätze und Präventionsstrategien dargestellt.

Im Unterschied zu HCV stehen für HBV seit Längerem zugelassene Impfstoffe zur Verfügung. *Brodzinski et al.* gehen in ihrem Beitrag der interessanten Frage nach soziodemografischen Determinanten in Deutschland der HBV-Infektion einerseits und der impfinduzierten Immunität andererseits nach. Sie beobachten eine Assoziation der HBV-Infektion bei Männern mit niedrigem Einkommen, bei Frauen mit niedriger Bildung. Umgekehrt ist die impfinduzierte Immunität bei Männern und Frauen mit höherem Einkommen und Bildung assoziiert.

Die Entwicklung des HBV-Impfstoffs stellt sicherlich einen Meilenstein in der Impfstoffentwicklung dar. *Wolfram Gerlich* beschreibt in seinem Beitrag die Entwicklung der verschiedenen Generationen der HBV-Impfstoffe und ihre hohe Wirksamkeit – einerseits eine Erfolgsgeschichte, andererseits bleiben dennoch Fragen offen, wie eine Rate von ca. 5 % sogenannter Non-Responder, die eine Weiterentwicklung des Impfstoffs nahelegt, bspw. durch Einbeziehung weiterer Epitope oder auch durch Anpassung an regional vorherrschende Subtypen.

Im Falle von HCV stellt die große genetische Variabilität eine wesentliche Herausforderung für die Entwicklung eines präventiven Impfstoffs dar. *Bankwitz et al.* beschreiben Determinanten einer schützenden Immunantwort gegen HCV. Dabei ist derzeit davon auszugehen, dass diese neben der Bildung neutralisierender Antikörper auch die robuste Induktion zytotoxischer T‑Zellen beinhalten muss. Die zunehmende Kenntnis über strukturelle und funktionelle Komponenten der HCV-spezifischen Immunantwort kann ein wesentlicher Impuls für die Entwicklung eines HCV-Impfstoffs sein.

Für HEV stehen in der EU noch keine zugelassenen Impfstoffe zur Verfügung. *Behrendt und Wedemeyer* gehen in ihrem Beitrag auf die Schwierigkeiten bei der Entwicklung eines sicheren Impfstoffs ein, der gegen die verschiedenen Genotypen von HEV wirksam ist. Sie stellen den Entwicklungsstand dreier verschiedener proteinbasierter Impfstoffe gegen HEV und deren Limitationen dar.

Die Relevanz von HEV für die öffentliche Gesundheit wurde auch in Deutschland lange nicht gesehen. *Johne et al.* beschreiben das zunehmende Auftreten von HEV-Fällen in Deutschland. Eine besondere Bedeutung kommt hier zoonotischen Übertragungen zu, insbesondere Wild- und Hausschweine stellen ein wesentliches Reservoir dar. Der Beitrag geht daher auch der Frage nach, welche Methodik geeignet ist, um sicherzustellen, dass durch Lebensmittel, die aus diesen Tieren hergestellt werden, keine Übertragung auf den Konsumenten erfolgt.

Während HBV und HCV schon seit Längerem als relevante Erreger hinsichtlich der Sicherheit von Blut und Blutprodukten anerkannt wurden, erfolgt die Testung auf HEV erst seit 2020. *Mitterreiter et al.* beschreiben in ihrem Beitrag die entsprechenden Regularien in Deutschland und die angewandte Methodik zum Nachweis von HBV, HCV und HEV. Dabei handelt es sich um Nucleinsäureamplifikationstechniken (NAT) und serologische Methoden, einerseits zum Nachweis viraler Antigene und andererseits zur Detektion humaner Antikörper gegen HBV, HCV und HEV.

Eine große Bedeutung hinsichtlich der Beratung zu spezifischen diagnostischen und klinischen Aspekten der Infektion mit Hepatitisviren kommt dem nationalen Referenzzentrum (NRZ) zu. *Glebe et al.* gehen in ihrem Beitrag auf die vielfältigen Aufgaben des NRZ für HBV und HDV in Gießen ein, wie beispielsweise die Initiation von Ringversuchen – auch im internationalen Kontext – zur Diagnostik der HBV-Resistenz oder die Entwicklung und Validierung von Referenzmaterialien. Das NRZ ist nun bereits seit 10 Jahren an der Universität in Gießen beheimatet.

Chronische Infektionen mit HBV und HCV sind in vielen Fällen mit einer ausgeprägten Pathogenese wie der Entwicklung einer Fibrose/Zirrhose oder eines hepatozellulären Karzinoms (HCC) verknüpft. *Glitscher et al.* beschreiben in ihrem Beitrag grundlegende Mechanismen der HBV- und HCV-assoziierten Pathogenese mit einem besonderen Fokus auf die Deregulation intrazellulärer Signaltransduktionswege und die Interferenz dieser Erreger mit zentralen Stoffwechselwegen.

Die häufig fatalen Verläufe chronischer Infektionen mit HBV, HCV und insbesondere auch HDV machen eine Therapie der chronischen Infektionen erforderlich. Eine Übersicht über die aktuell verfügbaren Therapien einer chronischen HBV-Infektion und eine Beschreibung ihrer Limitationen wird in dem Beitrag von *Neumann-Haefelin und Thimme* gegeben. Weiterhin werden neue Therapiestrategien, die derzeit in klinischer Entwicklung sind, vorgestellt und zukünftige Therapieansätze diskutiert.

Die Entwicklung der sog. DAAs („direct acting antivirals“) stellt einen wesentlichen Fortschritt in der Therapie der chronischen HCV-Infektion dar. *Peiffer und Zeuzem* erläutern in ihrem Beitrag die Wirkungsweise verschiedener DAAs, stellen den derzeitigen auf der Anwendung dieser DAAs basierenden interferonfreien Therapiestandard in Deutschland dar und beschreiben für unterschiedliche Patientengruppen die Viruseradikationsraten.

HDV als ein sog. Satellitenvirus, das die HBV-Hüllproteine zur Verbreitung nutzt, stellt im Vergleich zu den anderen im Themenheft behandelten viralen Hepatitiden eine Sonderform dar, gegen die lange Zeit keine spezifische Therapie verfügbar war. Myrcludex-Bulevirtide erhielt 2020 eine bedingte Zulassung von der Europäischen Arzneimittel-Agentur (EMA) zur Behandlung HDV-/HBV-co-infizierter Erwachsener. *Nkongolo et al.* beschreiben den Wirkmechanismus von Myrcludex-Bulevirtide, einem sog. Eintrittsinhibitor („entry inhibitor“), und die Ergebnisse verschiedener klinischer Studien, die einen deutlichen antiviralen Effekt auf HDV zeigen.

Aus Sicht des Herausgebers ist es in diesem Heft gelungen, Beiträge einer Vielzahl international ausgewiesener Expertinnen und Experten zu verschiedenen Aspekten der viralen Hepatitis zu gewinnen. Dies spiegelt auch die lange Tradition der klinischen und virologischen Hepatitisforschung in Deutschland wider. Den beteiligten Autorinnen und Autoren sei an dieser Stelle herzlich gedankt. So bleibt die Hoffnung, dass die in dem vorliegenden Heft vorgenommene Fokussierung auf ausgewählte Bereiche geeignet ist, einen Überblick über verschiedene Aspekte viraler Hepatitiden zu geben und die Neugier der Leserschaft zu wecken.

